# Independent erosion of conserved transcription factor binding sites points to shared hindlimb, vision and external testes loss in different mammals

**DOI:** 10.1093/nar/gky741

**Published:** 2018-08-23

**Authors:** Mark J Berger, Aaron M Wenger, Harendra Guturu, Gill Bejerano

**Affiliations:** 1 Department of Computer Science, Stanford University, Stanford, CA 94305-5329, USA; 2 Department of Electrical Engineering, Stanford University, Stanford, CA 94305-5008, USA; 3 Department of Developmental Biology, Stanford University, Stanford, CA 94305-5329, USA; 4 Department of Pediatrics, Stanford University, Stanford, CA 94305-5208, USA; 5 Department of Biomedical Data Science, Stanford University, Stanford, CA 94305-5464, USA

## Abstract

Genetic variation in *cis*-regulatory elements is thought to be a major driving force in morphological and physiological changes. However, identifying transcription factor binding events that code for complex traits remains a challenge, motivating novel means of detecting putatively important binding events. Using a curated set of 1154 high-quality transcription factor motifs, we demonstrate that independently eroded binding sites are enriched for independently lost traits in three distinct pairs of placental mammals. We show that these independently eroded events pinpoint the loss of hindlimbs in dolphin and manatee, degradation of vision in naked mole-rat and star-nosed mole, and the loss of external testes in white rhinoceros and Weddell seal. We additionally show that our method may also be utilized with more than two species. Our study exhibits a novel methodology to detect *cis-*regulatory mutations which help explain a portion of the molecular mechanism underlying complex trait formation and loss.

## INTRODUCTION

Genetic variation in *cis*-regulatory architecture is thought to be a major force in the morphological and physiological divergence of species (1–3). Although this hypothesis was initially controversial, growing experimental evidence suggests that gene regulation plays a key role in determining phenotypic traits (4,5), such as pelvic reduction in three-spined stickleback (6), loss of wing pigmentation in Drosophila (7) and loss of penile spines in human (8). These observations motivate studying the biological function of transcription factors, their target genes in different cellular contexts and the regulatory elements to which they bind (9). While recent studies have examined genome-wide patterns of evolution for transcription factor binding sites (10–12), assigning phenotypic traits to individual sites remains challenging.

Despite the conserved sequence-binding preferences of transcription factors, binding sites themselves can erode rapidly over the course of evolution (10,11). On average, individual binding sites are less conserved than the average protein coding gene (13,14) or enhancer (14). Additionally, binding site erosion does not appear to be offset by the creation of new binding events for the same transcription factor. For example, in the case of *CEBPA* and *HNF4A* binding events across five vertebrates, only half of the binding events lost in one lineage appear to be compensated by the creation of another binding event even within 10 kb of the original site (11). This rapid turnover of sites, along with the sheer number of binding events and lack of a clear genetic code, makes it particularly difficult to ascribe phenotypic function to transcription factor binding events (15).

**Figure 1.**
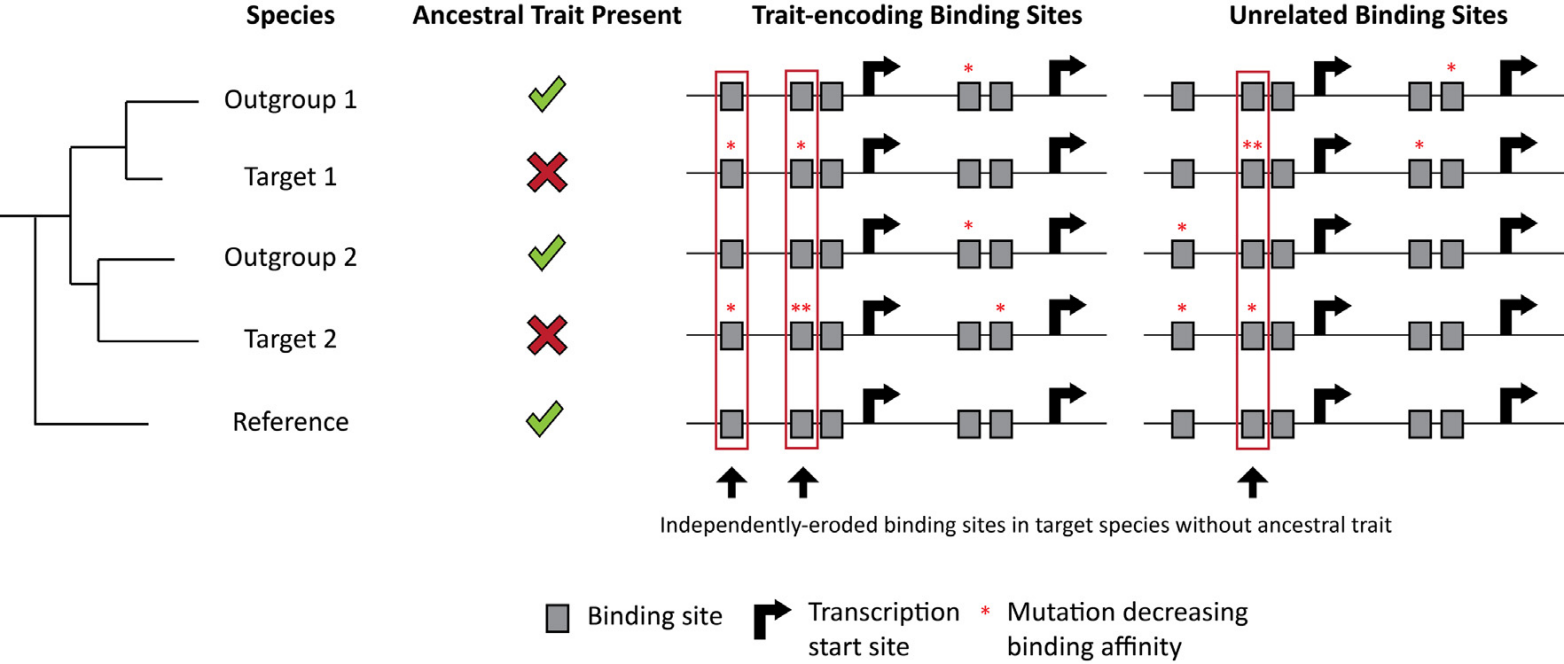
Given enough evolutionary time, independently eroded binding sites should congregate in genomic regions, which encode for independently lost traits. As species diverge, a phenotypic trait and the genomic regions required for that trait are inherited from the ancestral species. The trait of interest is necessary to maintain fitness, and therefore important trait-encoding transcription factor binding sites are under negative selection. As target species 1 evolves, a trait-loss event fixates within the species. However, the sister species outgroup 1 still maintains the trait. Since the trait is lost in target species 1, all trait-dedicated information now switches to neutral selection in the species. This leads to neutral erosion of trait-encoding transcription factor binding sites. Similarly, in target species 2, a trait-loss event (but not necessarily the same event as in target species 1) for the same trait fixates in the population. Here too, sister species outgroup 2 still maintains the trait. Now all trait-encoding information in target species 2 switch to neutral selection, and therefore the trait-encoding binding sites begin to neutrally erode. Using the sister species as outgroups, we can identify all transcription factor binding sites that have eroded in our target species but have been maintained in our outgroup species and many other references species. We refer to these sites as independently eroded binding sites. This very unusual evolutionary signature is shown in Figure 2 and Table 1 to be strongest next to key genes for the development of an important independently lost complex trait.

Despite their rapid turnover, individual binding sites (like individual amino acids) are known to be important building blocks in the formation of complex traits (16), motivating novel methodology of detecting putatively important binding events. Previous work has shown that independent loss of the same complex trait offers a great opportunity to find some of the genomic elements underlying complex trait formation (17,18). Based on those results, we were curious whether independently eroded highly conserved binding sites could also point to an independently lost trait. Conceptually, trait loss in independent clades should lead to neutral drift of all dedicated trait-encoding regions, regardless of the initial inactivating event (18). With sufficient evolutionary time, neutral erosion of these elements becomes detectable between species in which the trait differs. Therefore, in theory, eroded binding sites should congregate in genomic regions with a shared function relevant to an independent trait loss (Figure 1).

Using a large set of transcription factor binding site motifs, we set out to test this model by identifying binding sites that are eroded in two independent clades but are otherwise highly conserved across the phylogeny of placental mammals. For each pair of species, we performed a statistical test with 3538 ontology terms from the Mouse Genome Informatics (MGI) Gene Expression Database (GXD) (19) to determine the most significant shared biological function of the independently eroded transcription factor binding sites. Using our novel test, we recapitulate three diverse loss-of-function phenotypes over distinct clades, suggesting the generality of the approach.

## MATERIALS AND METHODS

### Multiple genome alignment and phylogenic tree

A hg19-anchored MULTIZ alignment of 100 species, along with the corresponding phylogenetic tree and branch lengths, was obtained from the UCSC Genome Browser (20) and subset to the provided 58 placental mammals (Supplementary Table S1 and Supplementary Figure S1). All analyses were performed on the MULTIZ alignment of 58 mammals. Exon and repeat annotations were obtained from the UCSC Genes and RepeatMasker tracks, respectively.

### Transcription factor binding motif library

The set of transcription factor binding motifs consists of 1154 unique high-quality monomer and dimer motifs for 753 transcription factors. Motifs were curated from existing databases and primarily literature as previously described (21). Both monomers and dimer motifs were included because previous studies have demonstrated that complexes have modified binding affinities (22,23).

### Identifying conserved transcription factor binding sites

Binding site prediction was performed on the multiple alignment of 58 Eutherian mammals using PRISM (24). PRISM performs binding site prediction by searching for motif-matching sequences, which are both conserved across multiple species and more conserved than their surrounding sequence, suggesting evolutionary constraint on the binding site. To identify these constrained sites, we first partitioned the reference genome into windows of conservation. Each base pair was assigned a weighted percent identity score by calculating the total branch length over which the base pair is conserved, normalized by the total branch length of the phylogeny. The percent identity score for each base was then smoothed by averaging the percent identity scores of a 100-bp window centered upon the base pair of interest. Conservation values over the entire multiple alignment were then grouped into 1% bins.

Next, for each binding motif, a set of null model motifs was generated by shuffling the columns of the position weight matrix. Adjacent CpG columns were conserved during shuffling. The shuffled motifs were then used to generate a set of predictions across the reference genome. Sequences with a MATCH (25) score of at least 0.8 were considered matches to the binding affinity matrix. For each 1% bin of conservation defined above, we recorded the distribution of Bayesian branch length (BBL) scores (26) for all sequences which match a shuffled motif. At last, the true motif was used to generate a set of predictions along the reference genome. For each prediction, we derived an empirical *P*-value based on the distribution of BBL scores for the respective conservation bin. Formally, given the distribution of scores for the respective conservation bin, the empirical *P*-value is the probability that a shuffled motif score is greater than or equal to the score of the real motif. A prediction was only retained if it had an uncorrected conservation *P*-value ≤10^−3^. Additionally, a prediction was only retained if the binding site is preserved in at least five species with a total phylogenetic branch length of 3.0 substitutions per site or more. Predictions were generated for the 58 placental mammals over all regions that did not contain an exon or a repeat element in the reference genome. See the PRISM paper for a more detailed explanation (24).

### Functional enrichment of predicted binding sites

To assess the validity of our binding site predictions, we asked whether PRISM binding sites are enriched for DNaseI hypersensitivity sites or regulatory histone modifications (H3K27ac, H3K4me1 and H3K4me3). All sites for genome assembly hg19 were downloaded from the ENCODE Project web portal (27). A global set of DNaseI and regulatory sites were formed by taking the union of all sites in each respective set using BEDTools (28).

Statistical significance was assessed using a permutation test. The top 5000 eroded binding sites were shuffled over all regions that did not contain an exon or a repeat element in the reference genome (the same regions examined by PRISM) using BEDTools (28). Shuffled regions were not allowed to overlap one another. The test statistic was the number of independently eroded binding sites, which overlap the set of regulatory regions. *P*-values were calculated as described in Phipson and Smyth (29).

### Detecting independently eroded sites

**Figure 2.**
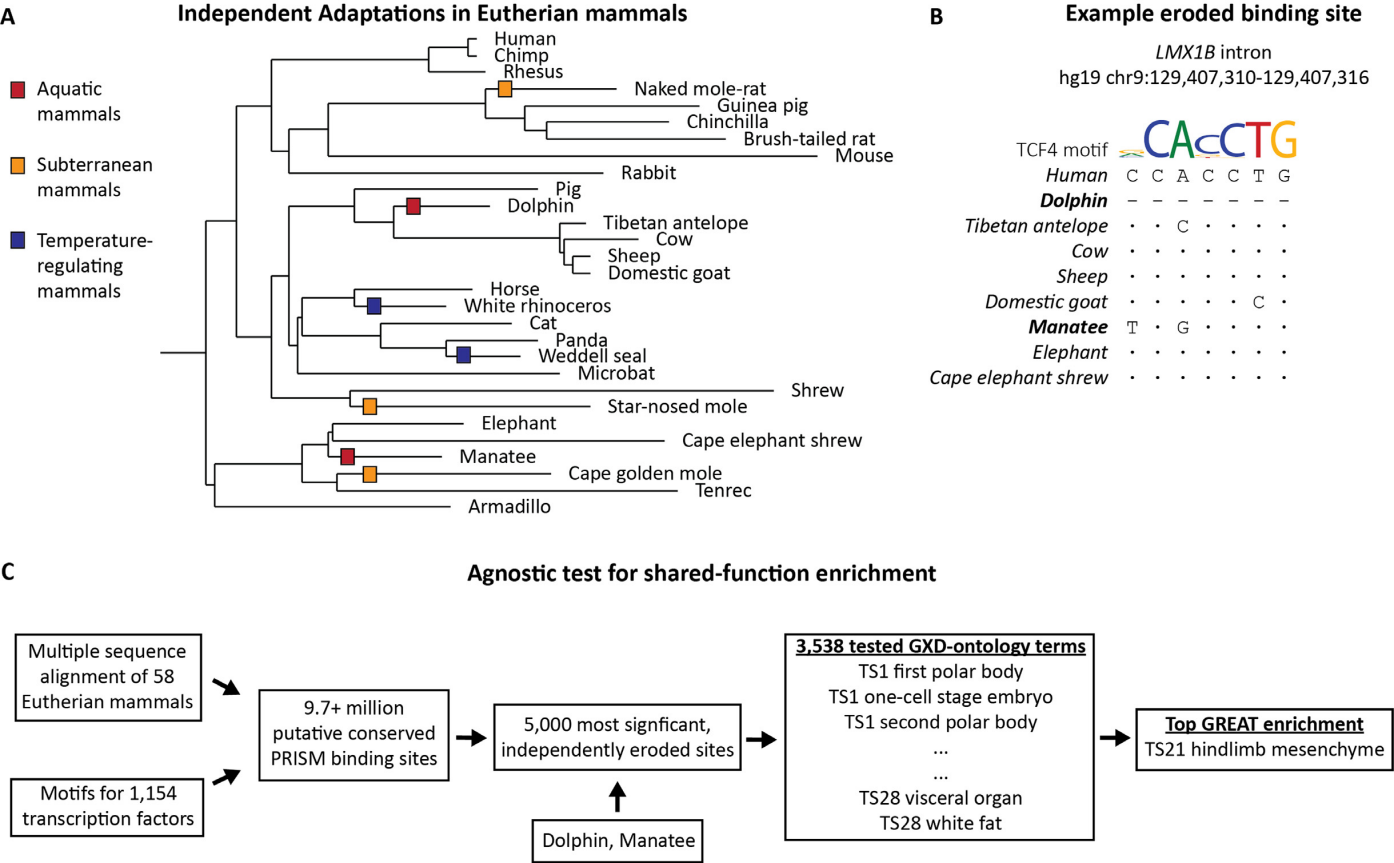
Independently eroded binding sites are enriched next to key genes for independent complex trait losses. (**A**) Throughout the evolution of placental mammals, species have independently adapted to a variety of environments. In the three pairs of species shown in a simplified phylogenic tree, we test whether independently eroded binding sites are statistically enriched for functions associated with these adaptations (see Supplementary Figure S1 for the phylogeny of all 58 mammals used in this study, and Figure 1 for the rationale). (**B**) Independently eroded binding sites can be the result of either a deletion (i.e. dolphin in this example) or mutations, which decrease binding affinity (i.e. manatee). Bases identical to human are represented as dots and single dashes represent deleted bases. (**C**) Using a library of 1154 transcription factor motifs, we identify ∼9.7 million putative mammalian conserved binding sites. We consider a conserved site to be eroded in the target species if a motif match is absent in the target species, but present in any of the outgroup species. Binding sites are considered independently eroded if they are eroded along two distinct clades of species. For a given pair of species, the 5000 most significant independently eroded sites are agnostically tested against 3538 ontology terms from the MGI Gene Expression Database to identify a most significant shared function (see Table 1).

For each species of interest, one or more outgroup species were dictated by the UCSC alignment as a reference to define a set of lineage-specific eroded sites. We selected the lowest common ancestor, which was thought to not exhibit the trait based on observations in extant species. All species derived from the least common ancestor that do not exhibit the trait of interest were used as the outgroup species (Figure 2A). We defined the eroded binding sites of a species to be the conserved binding site predictions that are present in any of the respective outgroup species (and therefore likely also in the ancestor), but not in the species of interest. Eroded site predictions overlapping assembly gaps were discarded (18).

### Inferring statistically significant accumulation of eroded binding sites near genes that share a common function

For a pair of target species, the eroded sites in each species were intersected to produce a set of independently eroded sites. These sites were ranked by their excess conservation score, and the top 5000 sites were used to form a high-confidence set of independently eroded sites. In order to prevent arbitrary tie-breaking, additional predictions were included if their excess conservation *P*-value was equivalent to the significance of the last prediction in the ranked list. Since paralogous transcription factors often have similar binding motifs, we required all entries in our set of most significant sites to not overlap one another. If multiple predictions did overlap, only the prediction with the highest excess conservation score was retained.

Each set of high-confidence independently eroded sites was submitted to the GREAT web portal (Genomic Regions Enrichment of Annotations Tool, http://great.stanford.edu) v3.0.0 (30). Binding sites were associated with target genes using the default ‘Basal plus extension’ association rule with the default distances of 5 kb upstream and 1 kb downstream from the canonical transcription start site, with an extension of up to 1 Mb or the next gene. The optimized thresholds from the original GREAT paper were used to define statistical significance: a region-based fold enrichment of at least 2, and a false-discovery rate (FDR) of ≤0.05 for both the region-based and gene-based tests. Analyses were conducted over the MGI Gene Expression Database (GXD) (19) for all terms *a priori* annotated with at least 10 genes and at most 500 genes, resulting in 3538 tests.

### Assessing tissue enrichment of the most significant eroded sites

For each pair of target species, we asked whether the top 5000 sites are also enriched for active regulatory elements in a phenotypically relevant tissue. When possible, active regulatory marks (H3K27ac, H3K4me1 and H3K4me3) were obtained from the ENCODE Project (27). If multiple ChIP-seq assays were not available, DNaseI open chromatin regions were obtained instead. When applicable, regions in mouse assembly mm10 were converted to hg19 using the liftOver tool from the UCSC Genome Browser (20). Statistical significance was assessed using a permutation test (see Functional enrichment of predicted binding sites).

### Identifying motif mismatches and deletions

At last, we asked whether motif mismatches or deletions are a more common mechanism for creating erosion events. To assess whether an erosion event is a mismatch or a deletion, we obtained the multiple alignment block(s) associated with the binding site prediction window used by PRISM (24). If any sequence aligned for the target species, we considered the erosion to be a motif mismatch disrupted by substitutions or small indels. Otherwise, if the target species does not have any aligning sequence, we consider the erosion event to be a deletion.

## RESULTS

### Loss of hindlimbs in aquatic mammals

Cetacean and Sirenian lineages have independently adapted to an aquatic habitat from their terrestrial ancestral species (31) (Figure 2A). Using the dolphin and manatee genomes as representative of each respective clade, we asked whether independently eroded binding sites in dolphin and manatee are associated with any morphological divergence of aquatic mammals.

To perform binding site prediction, we obtained an hg19-anchored MULTIZ alignment of 58 Eutherian mammals from the UCSC Genome Browser (20) (Supplementary Table S1). We also curated a set of 1154 high-quality transcription factor motifs from UniProbe (32), JASPAR (33) and TransFac (34). Both monomeric and dimeric motifs were included because previous work has demonstrated that complexes have modified binding affinities (22,23). With these resources, we used PRISM (24) to identify binding sites that are much more conserved than their surrounding sequence, suggesting evolutionary constraint and functional importance of the sites (see ‘Materials and Methods’ section). This method has been shown to substantially outperform conservation free position weight matrix prediction, as well as generate transcription factor binding profiles that are more similar to ChIP-seq data (24). In total, we identified 9 729 644 putative conserved transcription factor binding sites covering 0.848% of the reference genome (Figure 2C).

We then asked whether the predicted transcription factor binding sites are enriched for functional marks. We acquired all DNaseI and regulatory marks (H3K27ac, H3K4me1 and H3K4me3) data for human assembly hg19 from the ENCODE Project web portal (27). DNaseI and regulatory marks respectively covered 49.74% and 73.30% of the reference genome. PRISM binding sites are significantly enriched for both open chromatin (*P* = 9.99 × 10^−5^, fold = 1.31, permutation test with 10 000 permutations) and regulatory marks (*P* = 9.99 × 10^−5^, fold = 1.14, permutation test with 10 000 permutations), suggesting that the set of predicted transcription factor binding sites is enriched for functional regions of the genome.

Eroded transcription factor binding sites were defined as binding sites predicted in at least one outgroup species, but not in the target species of interest. For dolphin, the following species are present in the multiple alignment as the outgroup: Tibetan antelope, cow, sheep and domestic goat. For manatee, the multiple alignment offers elephant and cape elephant shrew as the outgroup species (Figure 2A and B). We discarded any eroded binding site prediction that overlapped an assembly gap in the target species (18). We identified 826 796 and 703 642 putative eroded binding sites in dolphin and manatee, respectively, with the intersection containing 80 798 independently eroded binding sites shared by both target species (Supplementary Table S2).

Previous work has identified human conserved non-coding elements (CNEs) that are independently lost in two mammalian lineages (17). We asked whether any of the independently eroded binding sites in our screen overlap these independently lost non-coding elements. Of the elements identified by Marcovitz *et al.* (17), 15 elements are independently lost in both dolphin and manatee. Only 15 of our 80 798 independently eroded transcription factor binding sites shared between these two species overlap the independently lost CNEs.

To assess the shared function of the most surprising erosion events, we selected the top 5000 most highly conserved sites (plus 11 ties at the cutoff point), according to PRISM (Supplementary Table S3). None of these sites overlapped previously identified independently lost CNEs (17). These binding sites were supplied to the GREAT web portal (Genomic Regions Enrichment of Annotations Tool, http://great.stanford.edu) (30) to agnostically determine the most significant shared function among the sites. Using GREAT’s default optimized settings, our analysis was conducted over 3538 ontology terms from the MGI Gene Expression Database (GXD) (19), a compendium of mouse developmental time points (Theiler stage, or TS1-28) with their corresponding validated expressed genes. The significance thresholds optimized in the original GREAT paper were used to define statistical significance: a region-based fold enrichment of at least 2 and a FDR of ≤0.05 for both the region-based and gene-based tests.

Strikingly, using these criteria, the most enriched term for dolphin and manatee set was ‘TS21 hindlimb mesenchyme’ with a region-based FDR of 1.3 × 10^−4^ and a region-based fold enrichment of 2.85 (Table 1 and Supplementary Table S4). Indeed, both cetaceans and sirenians have experienced a dramatic reduction or loss of hindlimbs (31,35–37). It is thought that the loss of hindlimbs contributes to streamlining the body, reducing drag and therefore drastically reducing the amount of energy necessary for swimming (38).

**Table 1.** Independently eroded binding sites congregate in the regulatory domain of important trait relevant genes

Parallel adaptations	Species	Top MGI expression term	Total eroded binding sites (genes)	False discovery rate (*Q*-value)	Fold enrichment	Affected target genes	Fraction of genes in top term affected
**Aquatic mammals**	Dolphin Manatee	**TS21 hindlimb mesenchyme**	37 (10)	1.30 × 10^−4^	2.85	*ADAMTS9, CDH11, FGF5, LIMK1, LMX1B, PITX2, RARG, SCXA, SCXB, ZEB1*	83.33%
‘The dolphin… develops hindlimb buds that do form an apical ectodermal ridge which regresses.’ (36)							
‘Manatees… retain only small pelvic rudiments and no external hindlimbs.’ (37)							
**Subterranean mammals**	Naked mole-rat Star-nosed mole	**TS21 optic stalk**	40 (9)	8.51 × 10^−7^	3.09	*GJA1, KCNH2, L1CAM, NRP1, OTX1, OTX2, PAX2, PRNP, PTPRZ1*	56.25%
‘[Naked mole-rats] have a degenerated eye and optic nerve, suggesting they have poor visual abilities.’ (42)							
**‘**Despite poorly developed vision, star-nosed moles make frequent and precise orienting movements to novel stimuli.’ (43)							
**Temperature-regulating mammals**	White rhinoceros Weddell seal	**TS18 mesonephric duct**	31 (11)	6.60 × 10^−4^	2.97	*C11orf34, GATA3, GDF11, KLF5, LAMA1, LAMA5, LAMB1, LAMC1, PAX2, SPINT2, WT1*	78.57%
‘During the evolutionary history of Laurasiatheria, the scrotum disappears… [in] Rhinocerotidae (rhinoceros)… [and] Phocidae (seals).’ (48)							
“Abnormalities of the [mesonephric] duct system are common in patients with cryptorchidism.” (47)							

See Figures 1 and 2 for the test we perform; TS, Theiler stage.

To corroborate our enrichment result, we asked whether the top 5000 binding sites are enriched for regulatory regions in the developing limb. H3K27ac, H3K4me1 and H3K4me3 marks were obtained from the ENCODE Project (27) for the developing mouse limb spanning time points E10.5 to E15.5. Regions were mapped from mouse assembly mm10 to hg19 using the liftOver tool from the UCSC genome browser (20). Merging the regions resulted in 182 805 contiguous regulatory regions spanning 10.90% of the reference genome. Of the 5011 most significant eroded sites, 1343 overlap limb regulatory regions (*P* = 9.99 × 10^−5^, fold = 2.64, permutation test with 10000 permutations). Additionally, 15 of the top 5000 independently eroded sites overlap 15 distinct experimentally verified VISTA enhancers (39), with 7 of the 15 being annotated as functional limb enhancers. Overall, our results identify a portion of the molecular signature underpinning the shared hindlimb feature and its subsequent loss.

### Degradation of vision in subterranean mammals

Both naked mole-rat and star-nosed mole have independently adapted to a subterranean habitat (Figure 2A). Using naked mole-rat and star-nosed mole as our target species, we asked whether independently eroded binding sites are associated with any morphological divergence of subterranean mammals.

For naked mole-rat, the following species were available as an outgroup: Guinea pig, chinchilla and brush-tailed rat. For the star-nosed mole, shrew served as the outgroup species (Figure 2A). Using the exact same methods from the aquatic mammals’ analysis above, we identified 969 015 and 797 304 putative eroded binding sites in naked mole-rat and star-nosed mole, respectively, with the intersection resulting in 105 416 independently eroded binding sites (Supplementary Table S2). Again, we selected the top 5000 most significant sites plus 121 ties (Supplementary Table S3). Using GREAT, the most enriched term for this set was ‘TS21 optic stalk’, a precursor structure to the optic nerve, with a region-based FDR of 8.51 × 10^−7^ and a region-based fold enrichment of 3.09 (Table 1 and Supplementary Table S4). Indeed, adaptation to a subterranean environment has led to regressive evolution of the visual system, with key vision-related genes eroded in these species (40–43).

We then asked whether the top 5000 sites are enriched for regulatory regions in the visual system. Due to a lack of epigenomic marks, we downloaded all DNaseI hypersensitivity sites for the mouse eye from the ENCODE Project (27). Regions were mapped from mouse assembly mm10 to hg19 using liftOver (20). Merging the accessible regions resulted in 390 140 contiguous regions spanning 2.99% of the reference genome. Of the 5121 most significant sites, 557 overlap open chromatin regions (*P* = 9.99 × 10^−5^, fold = 3.66, permutation test with 10 000 permutations). Additionally, 9 of the top 5000 sites overlap 10 distinct experimentally validated VISTA enhancers (39).

### Loss of external testes in mammals with temperature-regulating adaptations

Both phocid seals and rhinoceroses (Figure 2A) experience a wide range of external temperatures in their natural habitat. Both of these clades are able to maintain a relatively low body temperature compared to other Artiodactyla species (44), piquing interest in how both clades regulate their core body temperature (45,46). Using white rhinoceros and Weddell seal as the target species, we asked whether independently eroded binding sites are associated with any morphological divergence common to these temperature-regulating species.

For white rhinoceros, horse was available as the outgroup species. For Weddell seal, panda served as the outgroup species (Figure 2A). Using the previously described methods, we identified 447 297 and 425 706 putative eroded binding sites in white rhinoceros and Weddell seal, respectively, with the intersection resulting in 31 097 independently eroded binding sites (Supplementary Table S2). We selected the top 5000 most significant sites plus 102 ties (Supplementary Table S3). Using GREAT, the most enriched term for this set was ‘TS18 mesonephric duct’ with a region-based FDR of 6.6 × 10^−4^ and a region-based fold enrichment of 2.97 (Table 1 and Supplementary Table S4). The mesonephric duct is an embryonic tissue which forms the epididymis and vas deferens, both of which are commonly malformed in patients with cryptorchidism, the absence of testes from the scrotum (47). Indeed, both white rhinoceros and Weddell seal have lost external testes and therefore do not form a scrotum (48,49). Previous work speculates that the loss of external testes is in accordance with the development of an intra-abdominal gonadal cooling system in order to optimize sperm production (48).

We then asked whether the most significant eroded sites are enriched for regulatory regions in the testis. We downloaded regulatory marks (P300 and H3K4me3) for human testis from the ENCODE Project (27). Merging the regions resulted in 328 433 contiguous regions covering 3.64% of the reference genome. Of the 5102 most significant eroded sites, 287 lie in regulatory regions of the human testis (*P* = 9.99 × 10^−5^, fold = 1.57, permutation test with 10 000 permutations). Additionally, 11 of the top 5000 independently eroded sites overlap 10 distinct experimentally verified VISTA enhancers (39).

### Independently eroded binding sites tend to be created by transcription factor motif mismatches

We next asked whether independently eroded sites are more likely to arise from base pair mismatches or deletions of the ancestral sequence. For each set of the top 5000 independently eroded sites, we asked whether the ancestral sequence was disrupted by mutations or whether the ancestral sequence was deleted in each target species. For each pair of independently eroded sites, 58–66% are disrupted by motif matches in both species (Table 2). In contrast, only 6.8–8.9% of sites for each pair can be explained by two deletion events (Table 2). In accordance with existing work (11), our results suggest that transcription factor binding sites are more often disrupted by motif mismatches than deletion events.

**Table 2.** Motif mismatches are more common than sequence deletions in the creation of independently eroded transcription factor binding sites

Species (sites)	Both mismatches	One mismatch, one deletion	Both deletions
Dolphin & Manatee (5011)	3122	1510	379
Naked mole-rat & Star-nosed mole (5121)	2982	1682	457
White rhinoceros & Weddell seal (5102)	3384	1371	347

### Independently eroded binding sites can be utilized with additional species

At last, we asked whether the proposed method could be utilized for sets of three species. We explore two different scenarios: (i) the addition of a species that lies on the same clade and (ii) the addition of another species that lies on a distinct third clade.

To explore the use of an additional species that lies on the same clade, we revisited aquatic mammals. Killer whale is a sister species of dolphin and likely shares many of the same adaptations to an aquatic environment as dolphin and manatee (31). We asked whether conserved transcription factor binding sites that are independently eroded in dolphin, killer whale and manatee are associated with any morphological divergence of aquatic mammals.

For both dolphin and killer whale, the following species were utilized as an outgroup: Tibetan antelope, cow, sheep and domestic goat. For manatee, elephant and cape elephant shrew served as the outgroup species. Using the previously described methods, we found 826 796, 786 767 and 703 642 eroded sites in dolphin, killer whale and manatee, respectively. The intersection of the three sets resulted in 61 187 independently eroded sites (Supplementary Table S2). We selected the top 5000 most significant sites plus 50 sites (Supplementary Table S3). Using GREAT, the most enriched term was ‘TS21 hindlimb mesenchyme’ with region-based FDR of 4.24 × 10^−4^ and a region-based fold enrichment of 2.67 (Table 3 and Supplementary Table S4). The top 5000 most significant sites were also enriched for limb regulatory regions (*P* = 9.99 × 10^−5^, fold = 2.68, permutation test with 10 000 permutations). Indeed, all three species have undergone a drastic reduction or loss of hindlimbs (31,35).

**Table 3.** Independently eroded transcription factor binding sites congregate in the regulatory domains of important trait relevant genes using three species

Parallel adaptations	Species	Top MGI expression term	Total eroded binding sites (genes)	False discovery rate (*Q*-value)	Fold enrichment	Affected target genes	Fraction of genes in top term affected
**Aquatic mammals (same clade)**	Dolphin Killer whale Manatee	**TS21 hindlimb mesenchyme**	35 (9)	4.24 × 10^−4^	2.67	*ADAMTS9, CDH11, FGF5, LMX1B, PITX2, RARG, SCXA, SCXB, ZEB1*	75.00%
**Temperature-regulating mammals (distinct clade)**	White rhinoceros Weddell seal Dolphin	**TS22 mesonephros of male**	35 (7)	2.10 × 10^−18^	9.09	*DAZ1, DAZ2, DAZ3, DAZ4, FANCC, PTTG2, SPDYA*	53.84%

To explore the addition of another species that lies on a distinct third clade, we revisited temperature-regulating mammals. Dolphin lies on an independent clade from both white rhinoceros and Weddell seal, and is also known to regulate its core body temperature in accordance with the environment (50). We asked whether conserved transcription factor binding sites that are independently eroded in white rhinoceros, Weddell seal and dolphin are associated with any morphological divergence of temperature-regulating mammals.

For white rhinoceros and Weddell seal, the previously utilized species served as the outgroup species. For dolphin, Tibetan antelope, cow, sheep and domestic goat served as the outgroup species. Using the previously described methods, we identified 447 297, 425 706 and 826 796 eroded sites in white rhinoceros, Weddell seal and dolphin, respectively. The intersection of these three sets resulted in 7146 independently eroded binding sites. Selecting the most significant sites that do not overlap resulted in 2696 sites (Supplementary Table S3). Using GREAT, the most enriched term was ‘TS22 mesonephros of male’ with a region-based FDR of 2.10 × 10^−18^ and a region-based fold enrichment of 9.09 (Table 3 and Supplementary Table S4). These regions were not enriched for regulatory regions in human testis (*P* = 0.305, fold = 1.05, permutation test with 10 000 permutations). The male mesonephros is a precursor to the efferent ducts, which attaches to the rete testis and the epididymis (51). Similar to white rhinoceros and Weddell seal, dolphin also lacks external testes (48).

## DISCUSSION

The interpretation of *cis*-regulatory elements is a difficult task. Our inability to properly identify regulatory elements of interest inhibits the understanding of gene regulation in both evolution and disease. Here, we present a method to computationally identify highly conserved putative binding sites that are independently eroded in two clades of Eutherian mammals, and by extension are dedicated to trait encoding in the species that preserve both trait and sites. We show that for aquatic, subterranean and temperature-regulating mammals, the erosion of the highly conserved transcription factor binding sites respectively point to the following independent trait losses: loss of hindlimbs, degradation of vision and loss of external testes. Our results also demonstrate that our approach can be utilized with additional species on both the same and an additional clade. All tests are computed across 3538 ontology terms, demonstrating the statistical strength of our most-enriched terms. Additionally, our results further support the observation that motif mismatches are the more common mechanism for binding site erosion (11).

Our previous work linked independent erosion of protein-coding genes (18) and highly conserved enhancers (17) to independently eroded phenotypes. While these methods successfully identify genomic regions of large effects, current work suggests that complex traits are shaped by the accumulation of genomic events with small effect sizes (3,21,52). Therefore, studying the adaption of *cis*-regulatory regions at the resolution of individual transcription factor binding events is necessary to further our understanding of complex trait formation (21). While many binding events are rapidly gained and lost throughout the course of evolution (10,11), our test is able to successfully point to three diverse morphological changes, suggesting that analyses at the resolution of key individual binding sites are surprisingly tractable, likely not only across mammals but also across many different phyla.

**Figure 3.**
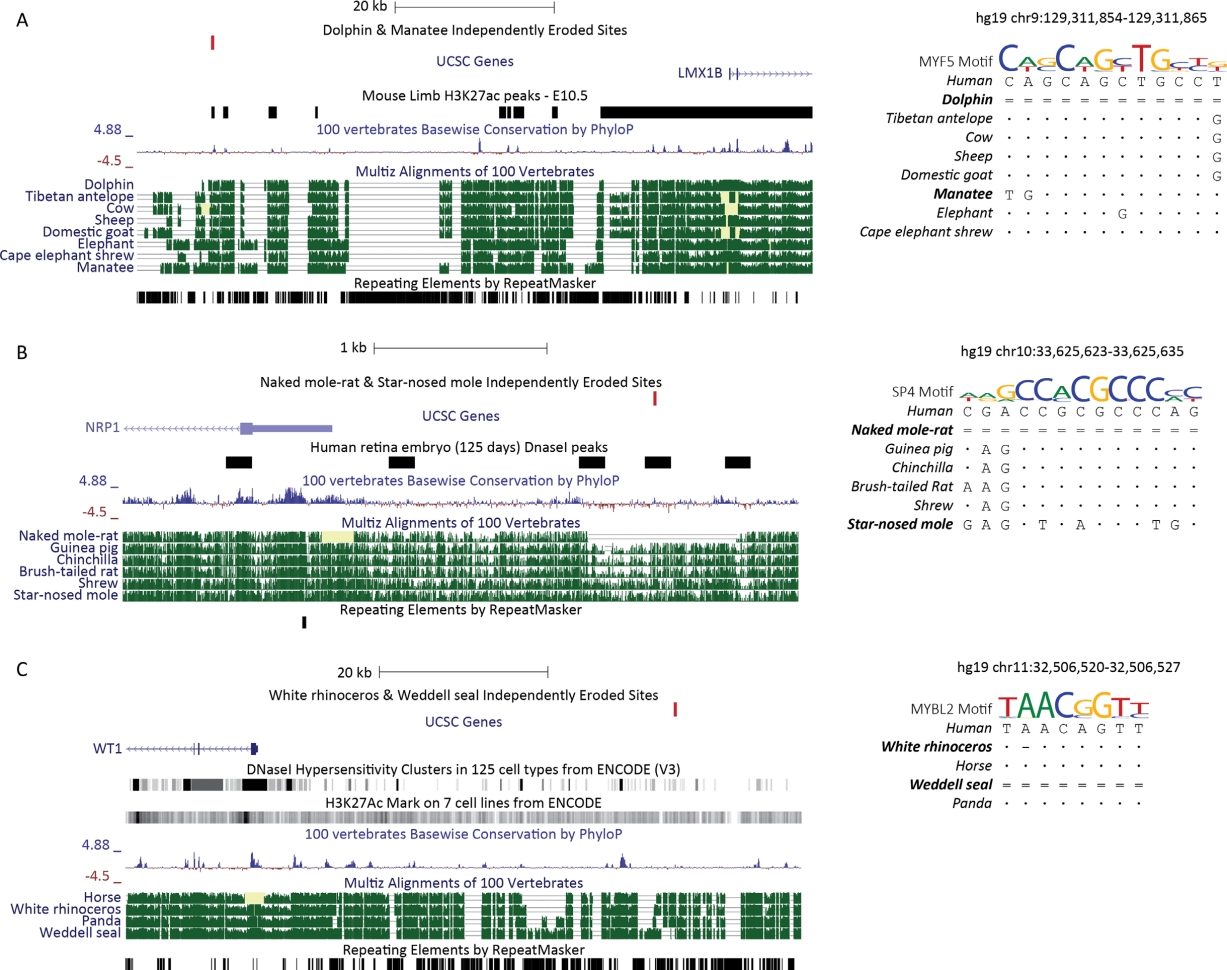
Examples of independently eroded binding sites next to important genes for independent complex trait loss. Bases identical to human are represented as dots, single dashes represent deletions and double dashes represent non-aligning bases at the query species. (**A**) In both dolphin and manatee, we find an independently eroded *MYF5* binding motif upstream of *LMX1B*. This binding site falls within an active mouse limb enhancer at E10.5 (27). *MYF5* is a myogenic transcription factor, the first myogenic factor to become active in the developing limb bud (59). *LMX1B* is a LIM homeodomain transcription factor responsible for dorsal cell fate in the developing limb (54,55). (**B**) In both naked mole-rat and star-nosed mole, we find an independently eroded *SP4* binding motif upstream of *NRP1*. This binding sites lies within a DNaseI peak in human retina embryo (125 days) (27). *SP4* is a transcription factor, which controls transcription of photoreceptor-specific genes in conjunction with *CRX* (60). *NRP1* is a transmembrane receptor necessary for proper angiogenesis and arteriogenesis of the retina (57). (**C**) In both white rhinoceros and Weddell seal, we find an independently eroded *MYBL2* binding motif upstream of *WT1*. This binding site intersects both a DNaseI hypersensitivity cluster and H3K27ac peaks from the ENCODE Project (27). *WT1* is a transcription factor involved in both renal and gonadal development. Conditional inactivation of *Wt1* in mice causes left-sided cryptorchidism with 40% penetrance (58).

Furthermore, our test recapitulates genes that are experimentally validated to be involved in the trait losses of interest (53–60). For aquatic mammals, the most enriched gene set includes *PITX2*, a homeobox transcription factor required for the formation of hindlimbs (53), and *LMX1B* (Figure 3A and Supplementary Figure S2A). *LMX1B* is a Lin-11, Isl-1, and Mec-3 (LIM) homeodomain transcription factor responsible for dorsal cell fate in the developing limb (54,55). For subterranean mammals, the most enriched ontology term includes *PAX2*, a transcription factor necessary for the development of the optic stalk (56). Rare germline mutations in *PAX2* are known to cause renal colomba syndrome, an autosomal-dominant disease characterized by optic nerve dysplasia (61). The most enriched term also includes *NRP1*, a transmembrane receptor necessary for proper angiogenesis and arteriogenesis of the retina (57) (Figure 3B and Supplementary Figure S2B). At last, for mammals with advanced temperature regulating capacity, the most enriched ontology term includes *WT1*, a transcription factor involved in both renal and gonadal development (Figure 3C and Supplementary Figure S2C). Patients with rare germline mutations in *WT1* have presented with cryptorchidism, the absence of testes from the scrotum (58). Previous work has also verified the association between *Wt1* and cryptorchidism using conditional gene inactivation in mice (58).

ChIP-seq, along with efforts such as the ENCODE Project (27), has transformed our ability to observe transcription factor binding. Despite these extensive resources, determining the phenotypic contribution of binding events is difficult. By identifying independently eroded binding sites and demonstrating that these sites point to independently lost morphological traits, we identify a small set of putative binding sites primed for experimental follow-up. As the research community sequences more genomes, characterizes the binding motifs of additional transcriptions factors and improves methods to model transcription factor binding, the ability to identify interesting binding events with our method will continue to improve. Coupled with the accuracy of CRIPSR/Cas genome-editing systems, our method enables the dissection of phenotypically relevant *cis*-regulatory regions to further biological understanding of complex-trait formation.

## DATA AVAILABILITY

Many extensions of our approach are possible, including the estimation of binding site erosion rates, an all-versus-all generalization of our screen and more. To facilitate such works, we provide conserved and eroded transcription factor binding sites for all 57 placental mammals, as well as code that allows picking species-specific erode sites, at http://bejerano.stanford.edu/tfbsErosion.

## Supplementary Material

Supplementary Data
