# Supplementary material for: Independent erosion of conserved transcription factor binding sites points to shared hindlimb, vision and external testes loss in different mammals
Source: Nucleic Acids Res. 2018 Aug 23;46(18):9299–308. doi: 10.1093/nar/gky741 (PMC6182171; doi:10.1093/nar/gky741)
Supplement: Supplementary Data [file gky741_supplemental_files.zip › si_legends.docx]

# **Full title**

Independent erosion of conserved transcription factor binding sites points to shared hindlimb, vision, and external testes loss in different mammals

# **Authors**

Mark J. Berger^1^, Aaron M. Wenger^1^, Harendra Guturu^2^, Gill Bejerano^1,3,4,5^

# **Affiliations**

^1^ Department of Computer Science, Stanford University, Stanford, CA, 94305-5329, USA

^2^ Department of Electrical Engineering, Stanford University, Stanford, CA, 94305-5329, USA

^3^ Department of Developmental Biology, Stanford University, Stanford, CA, 94305-5329, USA

^4^ Department of Pediatrics, Stanford University, Stanford, CA, 94305-5329, USA

^5^ Correspondence – [bejerano@stanford.edu](mailto:bejerano@stanford.edu)

# **Supplementary Information Legends**

## Supplementary Table 1

**Species List.** The list of 58 placental mammals and their respective genome assemblies used in this study.

## Supplementary Table 2

**All eroded transcription factor binding sites.** A full list of all the eroded transcription factor binding sites identified for each pair of species. Genomic coordinates are given relative to the reference genome (human, hg19).

## Supplementary Table 3

**Most significant independently eroded transcription factor binding sites.** A full list of the most significant independently eroded binding sites for each pair of species. Genomic coordinates are given relative to the reference genome (human, hg19). Coordinates highlighted in green are binding sites associated with the most enriched term (Table 1).

## Supplementary Table 4

**GREAT results for each set of most significant eroded binding sites.**

## Supplementary Figure 1

**Weighted phylogenetic tree.** A UCSC derived substitution per site weighted tree of the 58 species used in this study.

## Supplementary Figure 2

**Expanded context of eroded binding sites.** Binding sites from Figure 3 with a 100bp window around each site. The sequence depicted in Figure 3 is highlighted in red.
