# Supplementary figures and images for: Independent erosion of conserved transcription factor binding sites points to shared hindlimb, vision and external testes loss in different mammals
Source: Nucleic Acids Res. 2018 Aug 23;46(18):9299–308. doi: 10.1093/nar/gky741 (PMC6182171; doi:10.1093/nar/gky741)

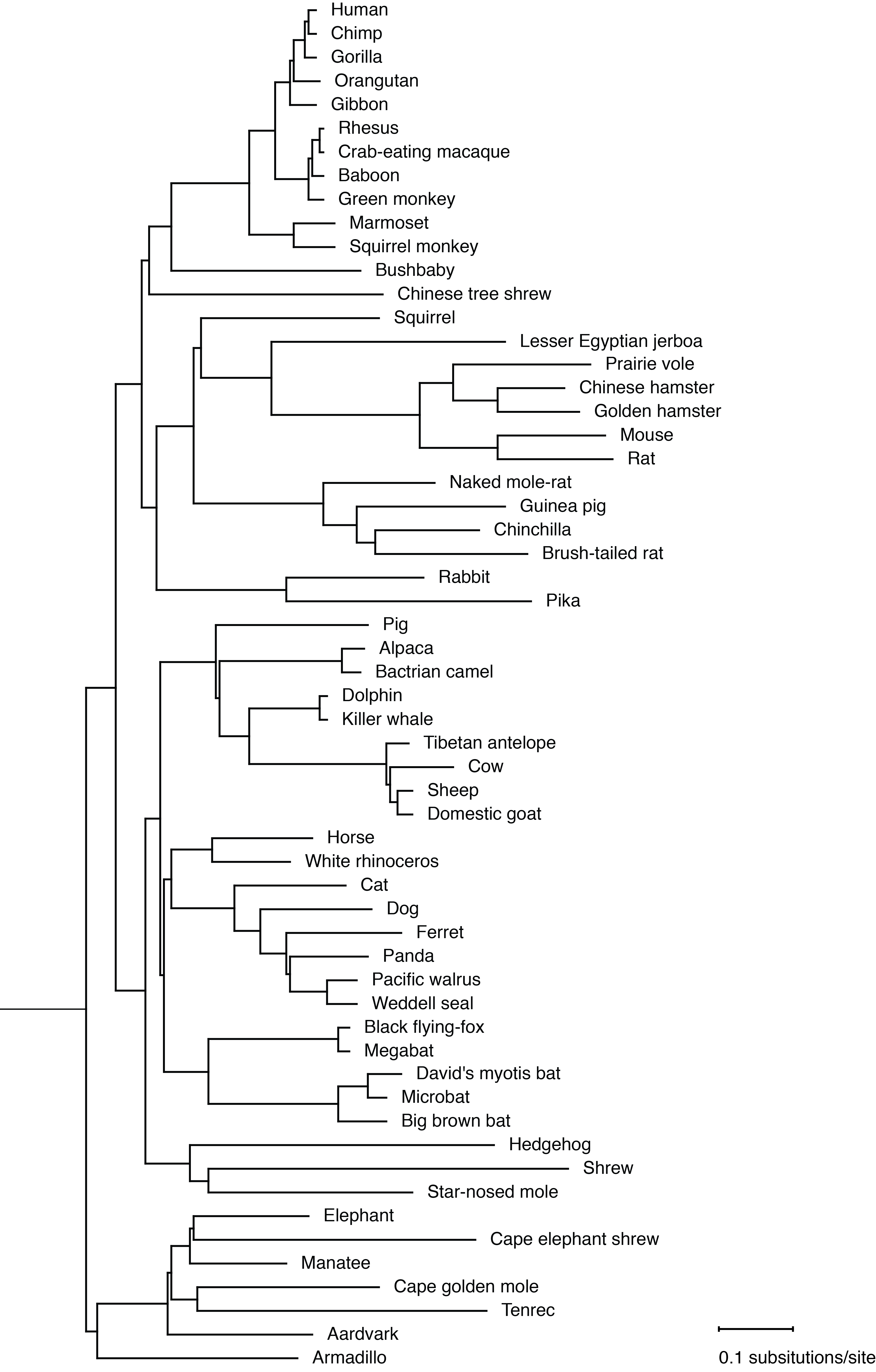

Supplement: Supplementary Data [file gky741_supplemental_files.zip › si_fig1.png]

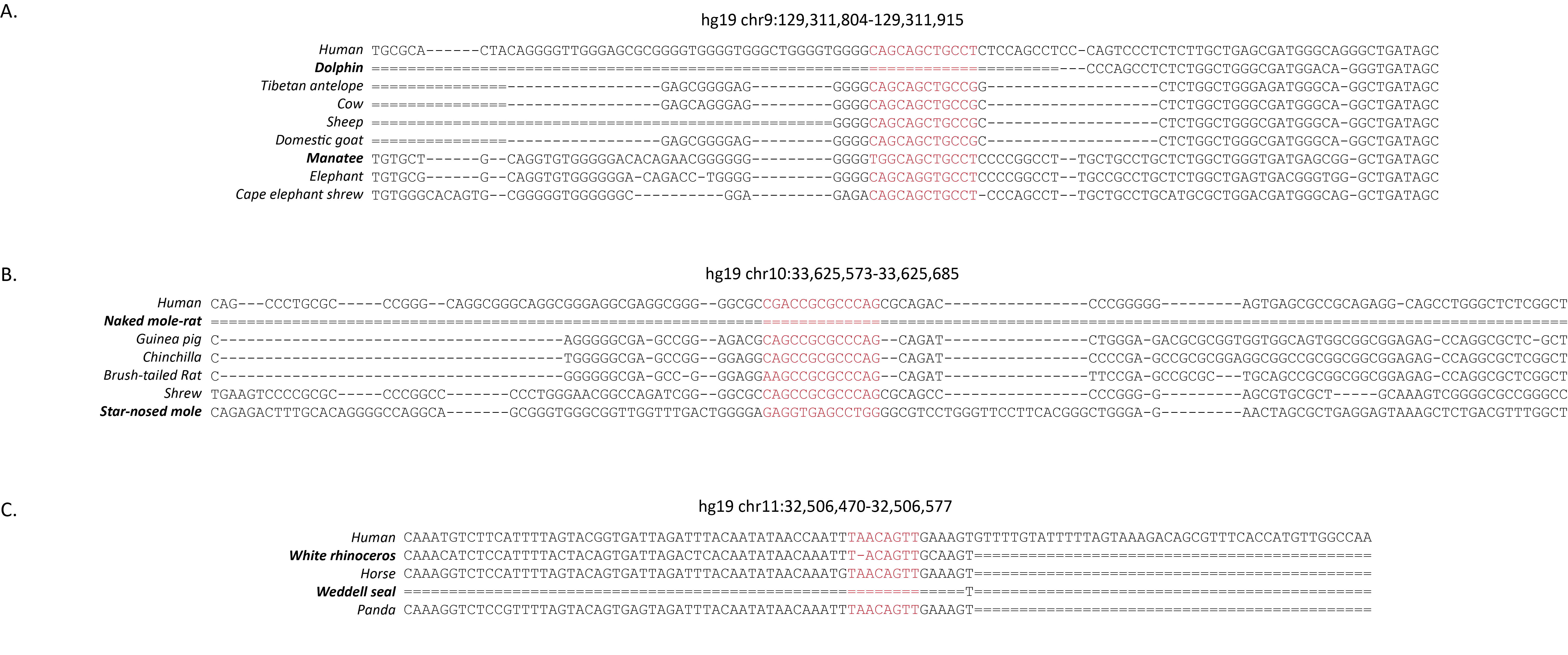

Supplement: Supplementary Data [file gky741_supplemental_files.zip › si_fig2.png]
